# Splicing factor mutations predict poor prognosis in patients with *de novo* acute myeloid leukemia

**DOI:** 10.18632/oncotarget.7000

**Published:** 2016-01-24

**Authors:** Hsin-An Hou, Chieh-Yu Liu, Yuan-Yeh Kuo, Wen-Chien Chou, Cheng-Hong Tsai, Chien-Chin Lin, Liang-In Lin, Mei-Hsuan Tseng, Ying-Chieh Chiang, Ming-Chih Liu, Chia-Wen Liu, Jih-Luh Tang, Ming Yao, Chi-Cheng Li, Shang-Yi Huang, Bor-Sheng Ko, Szu-Chun Hsu, Chien-Yuan Chen, Chien-Ting Lin, Shang-Ju Wu, Woei Tsay, Hwei-Fang Tien

**Affiliations:** ^1^ Division of Hematology, Department of Internal Medicine, National Taiwan University Hospital, Taipei, Taiwan; ^2^ Biostatistics Consulting Laboratory, Department of Nursing, National Taipei College of Nursing, Taipei, Taiwan; ^3^ Graduate Institute of Oncology, College of Medicine, National Taiwan University, Taipei, Taiwan; ^4^ Department of Laboratory Medicine, National Taiwan University Hospital, Taipei, Taiwan; ^5^ Tai-Chang Stem Cell Therapy Center, National Taiwan University, Taipei, Taiwan; ^6^ Clinical Laboratory Science and Medical Biotechnology, College of Medicine, National Taiwan University, Taipei, Taiwan; ^7^ Department of Pathology, National Taiwan University Hospital, Taipei, Taiwan

**Keywords:** de novo AML, splicing factor mutations, prognosis, paired sample

## Abstract

Mutations in splicing factor (SF) genes are frequently detected in myelodysplastic syndrome, but the prognostic relevance of these genes mutations in acute myeloid leukemia (AML) remains unclear. In this study, we investigated mutations of three SF genes, *SF3B1*, *U2AF1* and *SRSF2*, by Sanger sequencing in 500 patients with *de novo* AML and analysed their clinical relevance. SF mutations were identified in 10.8% of total cohort and 13.2% of those with intermediate-risk cytogenetics. SF mutations were closely associated with *RUNX1*, *ASXL1*, *IDH2* and *TET2* mutations. SF-mutated AML patients had a significantly lower complete remission rate and shorter disease-free survival (DFS) and overall survival (OS) than those without the mutation. Multivariate analysis demonstrated that SFmutation was an independent poor prognostic factor for DFS and OS. A scoring system incorporating SF mutation and ten other prognostic factors was proved very useful to risk-stratify AML patients. Sequential study of paired samples showed that SF mutations were stable during AML evolution. In conclusion, SF mutations are associated with distinct clinic-biological features and poor prognosis in *de novo* AML patients and are rather stable during disease progression. These mutations may be potential targets for novel treatment and biomarkers for disease monitoring in AML.

## INTRODUCTION

RNA splicing is a crucial post-transcription process that regulates gene expression and increases genomic diversity.[1] Recently, somatic mutations involving core components of the RNA splicing machinery were detected in myelodysplastic syndrome (MDS).[2, 3] Mutations of the splicing factor (SF) genes occur most frequently in *SRSF2, U2AF1*, and *SF3B1*, but also *ZRSR2, U2AF2, SF1, SF3A1, PRPF40B, PRPF8* and *LUC7L2*,[2] with a strong genotype and phenotype association.[4-6] Some of these mutations showed prognostic relevance in MDS, however, discrepancies exist among different studies.[7]

Although acute myeloid leukemia (AML) and MDS share some similar mutations in the pathogenesis, differences exist. For example, *NPM1*, *CEBPA* and *FLT3* mutations that are common in AML occur infrequently in MDS, and the opposite is true for *EZH2* and SF mutations. The reported incidence of SF mutations in AML varied from 4.5% to 12.5% depending on the patient population selected, the regions of SF genes screened, and the methods used.[2, 8-11] Due to lower incidence of SF mutations and small cohorts studied, the association of SF mutations with clinic-biologic features and their prognostic implication in *de novo* AML patients remain unclear. Further, there has been no report in literature concerning the stability of SF mutations in AML.

In this study, we assessed the clinical implication of SF mutations in 500 unselected adults with *de novo* AML and their interactions with other 18 genetic alterations. Longitudinal follow-ups of the status of SF mutations during the clinical course were also performed in 163 patients to investigate the stability and pathogenic role of these mutations in AML. To the best of our knowledge, this is the first study to address the prognostic implication of SF mutations in a large cohort of patients with *de novo* AML. We found that SF mutation was an independent poor-risk factor for overall survival (OS) and disease-free survival (DFS) in these patients.

## RESULTS

### SF mutations in patients with *de novo* AML

**Figure 1:**
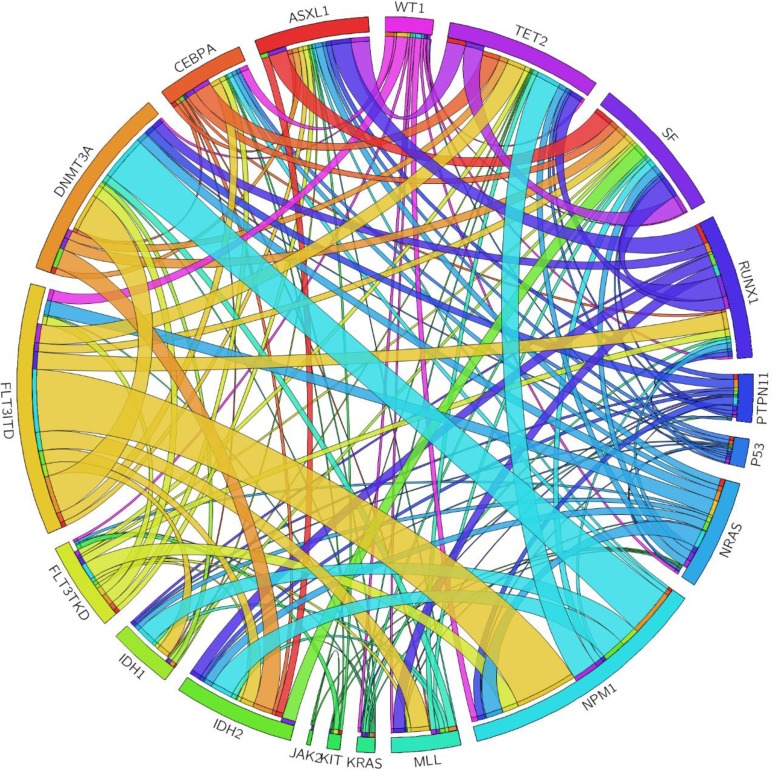
The Circos plots depicted the relative frequency and pairwise co-occurrence of genetic alterations The length of the arc corresponded to the frequency of the first gene mutation, and the width of the ribbon corresponded to the proportion of the second gene mutation.

Mutations of the RNA splicing machinery genes were identified in 54 (10.8%) of 500 patients, including 12 (2.4%) with *SF3B1* mutations, 15 (3.0%) with *U2AF1* mutations, and 27 (5.4%) with *SRSF2* mutations, respectively (Table 1). SF mutations in all these patients were heterozygous. None had two of these three SF mutations at the same time, suggesting these three mutations were mutually exclusive (Table 1, Figure 1 and Supplementary Table 4).

The most common *SF3B1* mutation was K666M (*n* = 5), followed by K700E (*n* = 3) (Table 1). Ring sideroblasts could be detected in two (33%) of the six patients who had bone marrow smears for iron staining. Regarding *U2AF1* mutations, ten patients had exon 2 mutations, including S34F in six patients, S34Y in three and S34T in one; six patients had exon 5 mutations, including Q157P in three patients, Q157R in two and E159_M160insYE in one. One patient (patient 18) had concurrent exon 2 S34F and exon 6 Q157R mutations. Among the 27 *SRSF2-*mutated patients, 24 patients had missense mutations, including P95H in 12 patients, P95L in 8 and P95R in 4. Two patients (patients 28 and 54) had P95_R102del (c.284_307del), a 24-base pair deletion, and the remaining one patient (patient 48) had R94_p95insR (c.283_284insGCC), a 3-base pair insertion (Table 1).

**Table 1:** The mutation patterns in 54 patients with SF3B1/U2AF1/SRSF2 mutations at diagnosis

UPN	Age/Sex	FAB	RNA Splicing mutation			Other accompanied gene mutations
			Location	DNA change	Protein change	
SF3B1 (n=12)						
1	65/F	1	Exon 15	c.2112_2117dup	S705_A706dup	*NRAS, ASXL1*
2	77/M	1	Exon 14	c.1998G>C	K666N	*RUNX1, DNMT3A*
3	67/F	1	Exon 14	c.1998G>C	K666N	*RUNX1, TET2, P53*
4	53/F	1	Exon 14	c.1998G>C	K666N	*CEBPA, RUNX1*
5	73/F	1	Exon 15	c.2098A>G	K700E	*CEBPA, TET2, DNMT3A*
6	62/M	2	Exon 14	c.1996A>C	K666Q	*FLT3*/ITD, *MLL*/PTD, *RUNX1*
7	70/M	4	Exon 14	c.1988C>T	T663I	*NPM1*
8	31/M	3	Exon 14	c.1998G>C	K666N	—
9	43/F	1	Exon 15	c.2098A>G	K700E	*P53*
10	82/M	4	Exon 14	c.1998G>C	K666N	*FLT3*/ITD, *MLL*/PTD, *RUNX1*
11	86/M	5	Exon 14	c.1873C>T	R625C	*FLT3*/ITD, *DNMT3A*
12	70/M	2	Exon 15	c.2098A>G	K700E	*NPM1*, *FLT3*/ITD, *DNMT3A, IDH2*
U2AF1 (n=15)						
13	40/M	1	Exon 2	c.101C>T	S34F	*IDH2*
14	22/M	4	Exon 6	c.470A>G	Q157R	*FLT3*/TKD, *ASXL1*
15	54/M	4	Exon 2	c.101C>A	S34Y	*PTPN11, ASXL1, DNMT3A*
16	75/M	4	Exon 2	c.101C>T	S34F	*KRAS*
17	72/M	1	Exon 6	c.470A>C	Q157P	*ASXL1, IDH1, TET2*
18	52/M	0	Exon 2	c.101C>T	S34F	*ASXL1*
			Exon 6	c.470A>G	Q157R	
19	47/M	4	Exon 2	c.101C>A	S34Y	*PTPN11*
20	43/M	2	Exon 2	c.101C>T	S34F	*WT1*
21	71/F	2	Exon 2	c.101C>T	S34F	*CEBPA, NRAS, TET2*
22	66/M	2	Exon 6	c.476_477insGTATGA	E159_M160insYE	*NRAS, IDH2*
23	47/M	1	Exon 2	c.101C>T	S34F	*—*
24	48/M	6	Exon 6	c.470A>C	Q157P	*RUNX1*
25	44/F	0	Exon 2	c.101C>T	S34T	—
26	46/M	2	Exon 2	c.101C>A	S34Y	*FLT3*/ITD, *MLL*/PTD
27	71/F	4	Exon 6	c.470A>C	Q157P	*NRAS, IDH2*
SRSF2 (n=27)						
28	89/M	4	Exon 2	c.284_307del	P95_R102del	*RUNX1, IDH2*
29	80/M	U	Exon 2	c.284C>T	P95L	—
30	71/M	4	Exon 2	c.284C>A	P95H	*TET2*
31	73/M	U	Exon 2	c.284C>G	P95R	—
32	67/M	U	Exon 2	c.284C>A	P95H	*ASXL1, IDH2*
33	85/F	2	Exon 2	c.284C>T	P95L	*CEBPA, ASXL1, TET2*
34	66/M	2	Exon 2	c.284C>A	P95H	*NPM1, RUNX1, ASXL1*
35	70/M	5	Exon 2	c.284C>A	P95H	*ASXL1, TET2*
36	65/M	1	Exon 2	c.284C>G	P95R	*IDH1*
37	64/M	2	Exon 2	c.284C>T	P95L	*CEBPA, IDH2*
38	42/F	4	Exon 2	c.284C>A	P95H	*FLT3/ITD, RUNX1, ASXL1, DNMT3A*
39	75/M	5	Exon 2	c.284C>A	P95H	*NPM1, ASXL1, TET2*
40	84/M	0	Exon 2	c.284C>T	P95L	*RUNX1, IDH2, DNMT3A*
41	68/M	4	Exon 2	c.284C>T	P95L	*RUNX1, TET2*
42	66/M	4	Exon 2	c.284C>A	P95H	*NRAS, ASXL1*
43	63/M	2	Exon 2	c.284C>A	P95H	*NRAS, TET2*
44	72/M	1	Exon 2	c.284C>G	P95R	*RUNX1, IDH1*
45	82/M	5	Exon 2	c.284C>T	P95L	*RUNX1, ASXL1, TET2*
46	70/M	4	Exon 2	c.284C>A	P95H	*KRAS, RUNX1*
47	48/F	1	Exon 2	c.284C>A	P95H	*CEBPA, IDH2, DNMT3A*
48	71/M	1	Exon 2	c.283_284insGCC	R94_p95insR	*RUNX1, TET2*
49	77/F	4	Exon 2	c.284C>A	P95H	*NPM1, TET2*
50	87/M	2	Exon 2	c.284C>T	P95L	*NPM1*, *FLT3*/ITD, *TET2*
51	63/F	4	Exon 2	c.284C>G	P95R	*PTPN11, IDH2*
52	90/M	2	Exon 2	c.284C>T	P95L	*CEBPA, ASXL1, TET2, P53*
53	69/M	4	Exon 2	c.284C>A	P95H	*NRAS, FLT3*/TKD, *RUNX1, IDH2, DNMT3A*
54	44/M	1	Exon 2	c.284_307del	P95_R102del	*RUNX1, IDH2*

Abbreviations: UPN, unique patient number; FAB, French-American-British; U, undetermined.

### Correlation of SF mutations with clinical and laboratory features

SF-mutated patients were older (median, 67.5 years *vs.* 49 years, *P* < 0.0001, Table 2), male predominant (14.4% in males *vs.* 6% in females, *P* = 0.0033) and had a lower incidence of FAB M2 subtype (*P* = 0.0499) than other patients. The SF mutations were positively associated with the expression of HLA-DR (*P* = 0.0156) and CD34 (*P* = 0.0131), but inversely associated with the expression of CD33 (*P* = 0.0379) and CD56 (*P* = 0.0493) on the leukemic cells (Supplementary Table 1). Correlation of the clinical and laboratory features with mutations in individual SF genes was shown in Supplementary Table 2.

**Table 2:** Comparison of clinical and laboratory features between AML patients with and without SF mutation

Variables	Total (*n* = 500)	SF-Mutated (*n* = 54, 10.8%)	SF-Wild (*n* = 446, 89.2%)	*P* value
**Sex**^†^				0.0033
Male	285	41 (14.4)	244 (85.6)	
Female	215	13 (6)	202 (94)	
**Age (year)**^‡^	51 (15-90)	67.5 (22-90)	49 (15-90)	<0.0001
**Lab data**^‡^				
WBC (/μL)	19075 (120-627800)	19865 (120-627800)	19090 (300-42300)	0.9837
Hb (g/dL)	8 (2.9-16.2)	8.2 (3.7-16.2)	8 (2.9-14)	0.5309
Platelet (×1,000 /μL)	42 (2-802)	36.5 (6-455)	42 (2-802)	0.6565
Blast (/μL)	7401 (0-456725)	6212 (14-456725)	7479 (0-369070)	0.953
LDH (U/L)	889 (206-15000)	821 (288-7930)	856 (206-15000)	0.8432
**FAB**^†^				
M0	10	3 (30)	7 (70)	0.0827
M1	112	14 (12.5)	98 (87.5)	0.4933
M2	171	12 (7.0)	159 (93.0)	0.0499
M3	38	1 (2.6)	37 (97.4)	0.1058
M4	124	16 (12.9)	108 (87.1)	0.4052
M5	24	4 (16.7)	20 (83.3)	0.3139
M6	12	1 (8.3)	11 (91.7)	>0.9999
Undetermined	9	3 (33.3)	6 (66.7)	0.0626
Induction response*	363	32	331	
CR	284	11 (34.4)	273 (82.5)	<0.0001
PR/Refractory	54	15 (46.9)	39 (11.8)	<0.0001
Induction death	25	6 (18.7)	19 (5.7)	0.0153
Relapse*	144	7 (63.6)	137 (50.2)	0.5412

† number of patients (%)

‡ median (range)

* only the 363 patients, including 32 with SF mutation and 331 without, who received conventional intensive induction chemotherapy and then consolidation chemotherapy if CR was achieved, as mentioned in the text, were included in the analysis.

### Association of SF mutations with cytogenetic abnormalities

Chromosome data were available in 482 patients at diagnosis, including 51 SF*-*mutated and 431 SF-wild patients (Supplementary Table 3). SF mutations occurred more frequently in patients with intermediate-risk cytogenetics (13.2%) than in those with favorable- or unfavorable-risk cytogenetics (5.5%, *P* < 0.0001). None of the patients with t(8;21), inv(16), or t(7;11) showed SF mutation, but one patient with t(15;17) harbored this mutation concurrently. There was no association of SF mutations as a whole with other chromosomal abnormalities, including +8, +11, +13, +21, -5/del(5q), and -7/del(7q). Intriguingly, *U2AF1* mutations occurred frequently in patients with -7/7q-(*P* = 0.0352)

### Association of SF mutations with other molecular gene abnormalities

The interaction of SF mutations with mutations of 18 other genes was shown in Table 3. Among the 54 patients with SF mutations, 49 (90.7%) showed additional molecular abnormalities at diagnosis (Tables 1 and 3 and Figure 1). Eleven had one additional change, 21 had two, 12 had three, four had four and one had five. Patients with SF mutations had significantly higher incidences of *RUNX1*, *ASXL1*, *IDH2* and *TET2* mutations than those without the mutation (31.5% *vs.* 10.1%, *P* < 0.0001; 27.8% *vs.* 7.8%, *P* < 0.0001; 20.4% *vs.* 9.9%, *P* = 0.0344 and 27.8% *vs.* 11.4%; *P* = 0.0022, respectively). The interaction of mutations in each SF gene and other genetic alterations was shown in Supplementary Table 4.

**Table 3:** Association of SF mutation with other gene mutations

Variables	No. of patients with alteration (%)			*P* value
	Whole cohort (*n* = 500)	SF-mutated patients (*n* = 54)	SF-wild patients (*n* = 446)	
*FLT3/*ITD	113 (22.6)	7 (13.0)	106 (22.7)	0.0848
*FLT3/*TKD	38 (7.6)	2 (3.7)	36 (8.1)	0.4116
*NRAS*	61 (12.2)	7 (13.0)	54 (12.1)	0.8266
*KRAS*	16 (3.2)	2 (3.7)	14 (3.1)	0.6874
*PTPN11*	18 (3.6)	3 (5.6)	15 (3.3)	0.4291
*KIT*	15 (3.0)	0 (0)	15 (3.3)	0.3891
*JAK2*	3 (0.6)	0 (0)	3 (0.7)	>0.9999
*WT1*	33 (6.6)	1 (1.9)	32 (7.2)	0.239
*NPM1*	103 (20.6)	6 (11.1)	97 (21.7)	0.0753
*CEBPA*	66 (13.2)	7 (13.0)	59 (13.2)	>0.9999
*RUNX1*	62 (12.4)	17 (31.5)	45 (10.1)	<0.0001
*MLL/*PTD	27 (5.4)	3 (5.6)	24 (5.4)	>0.9999
*ASXL1*	50 (10.0)	15 (27.8)	35 (7.8)	<0.0001
*IDH1*	27 (5.4)	3 (5.6)	24 (5.4)	>0.9999
*IDH2*	55 (11)	11 (20.4)	44 (9.9)	0.0344
*TET2*	66 (13.2)	15 (27.8)	51 (11.4)	0.0022
*DNMT3A*	70 (14.0)	9 (16.7)	61 (13.7)	0.5353
*TP53*	35 (7.0)	2 (3.7)	33 (7.4)	0.409

### Impact of SF mutation on response to therapy and clinical outcome

**Figure 2:**
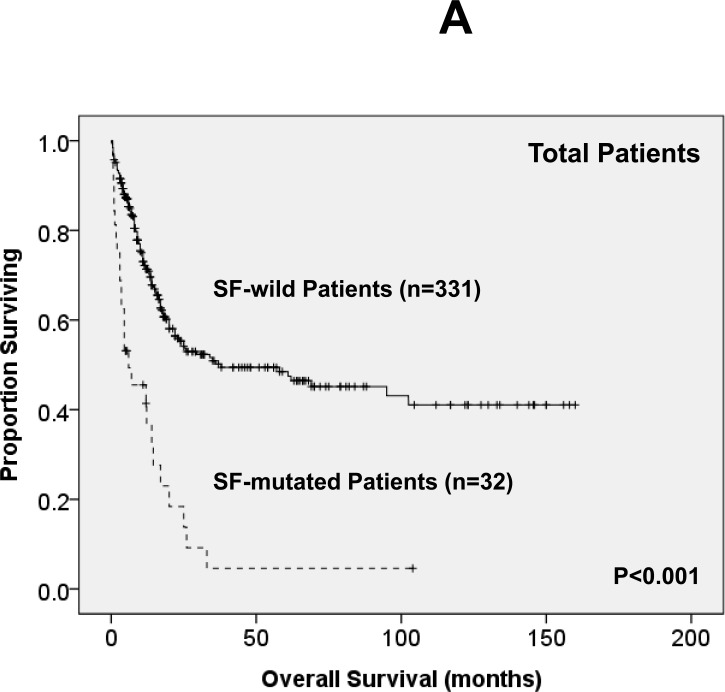

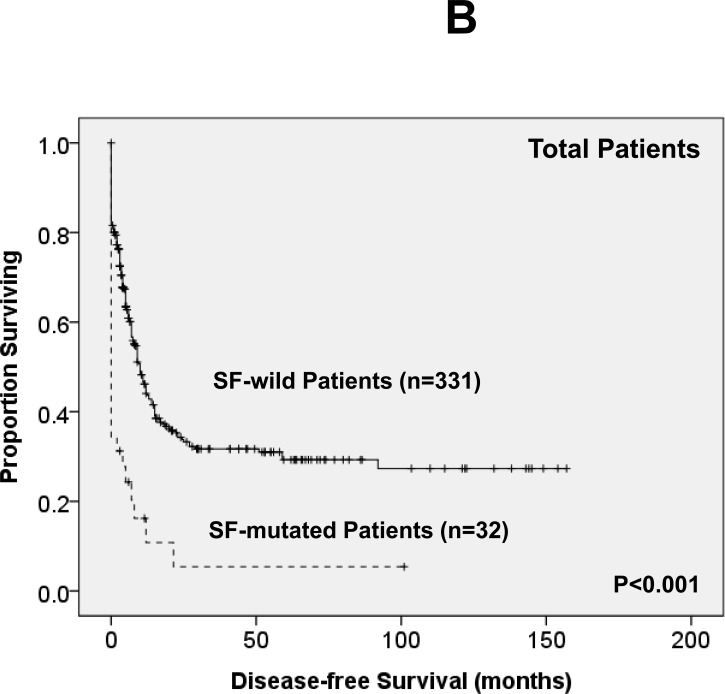

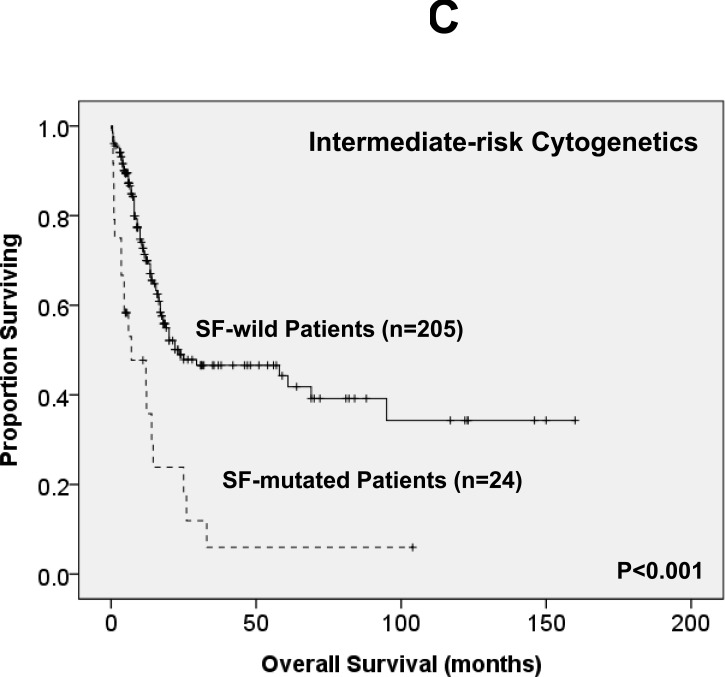

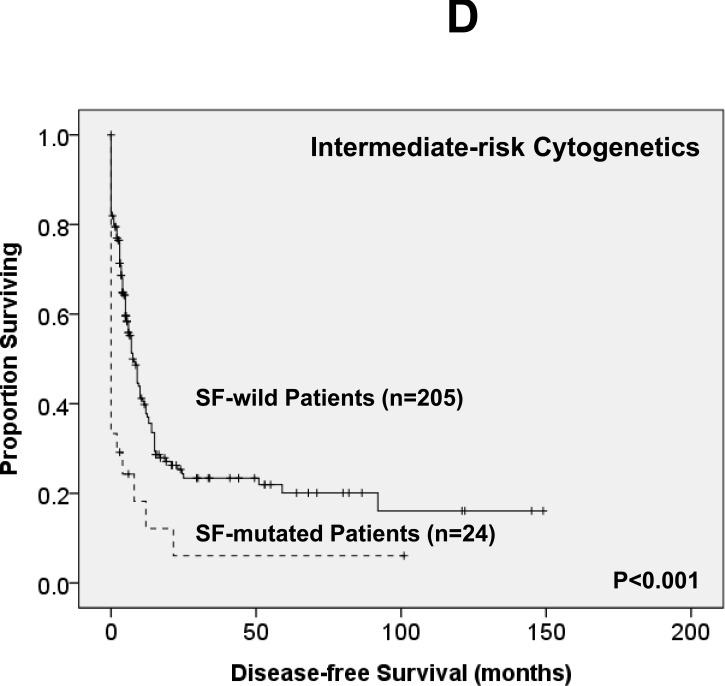

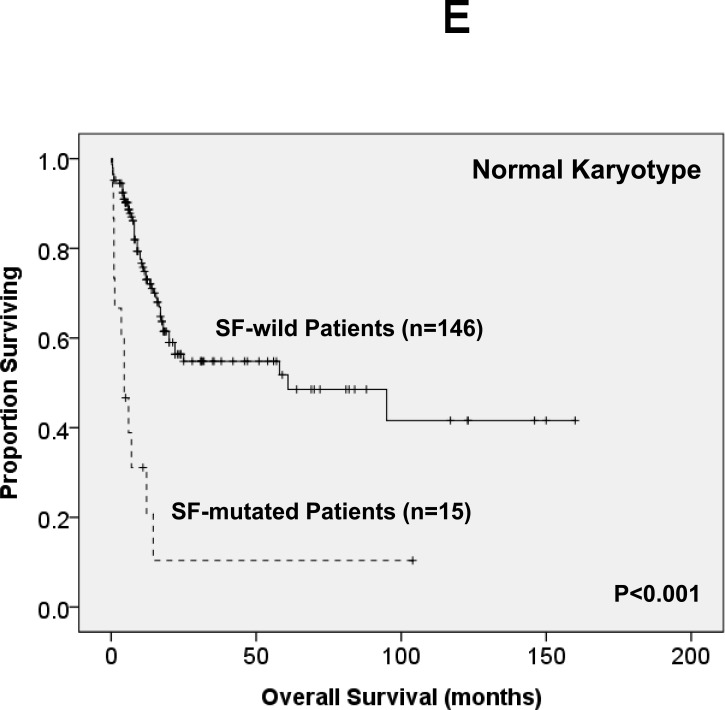

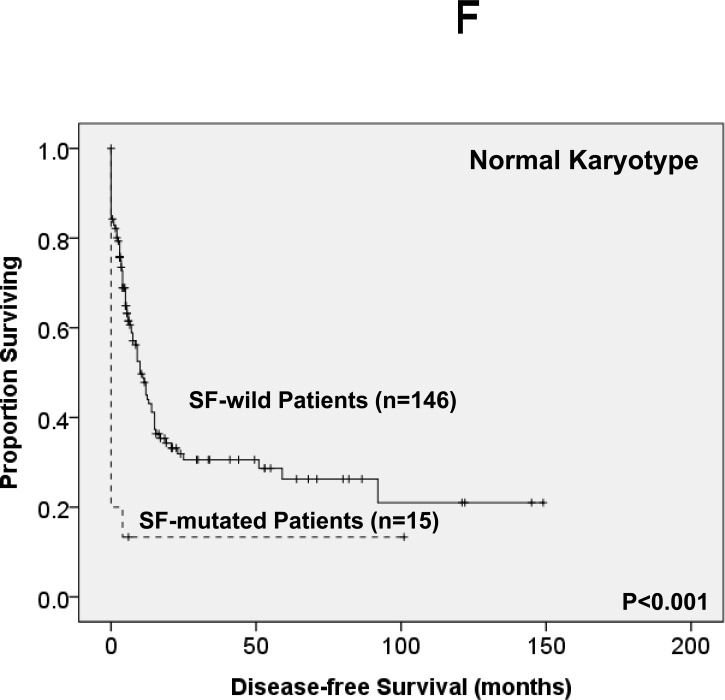
Kaplan-Meier survival curves for overall survival and disease-free survival stratified by the status of SF mutations in total 363 AML patients (A and B), 229 patients with intermediate-risk cytogenetics (C and D) and 161 patients with normal karyotype (E and F) who received standard intensive chemotherapy

Of the 363 AML patients undergoing conventional intensive induction chemotherapy, 284 (78.5%) patients achieved a CR. Mutations in any of *SF3B1*, *SRSF2* and *U2AF1* were associated with lower CR rates (22.2% *vs*. 79.7%, *P* = 0.0005; 45.5% *vs*. 79.3%, *P* = 0.0162; 33.3% *vs*. 79.8%, *P* = 0.0009; respectively, Supplementary Table 2A, B, C). With a median follow-up of 55 months (ranges, 1.0 to 160), patients with mutations of either *SF3B1* or *U2AF1* had significantly shorter OS (2 months *vs*. 29.5 months, *P* < 0.001 and 4.5 months *vs*. 26 months, *P* = 0.001, respectively, Supplementary Figure 1A, E) and DFS (0 month *vs*. 9 months, *P* < 0.001 and 0 month *vs*. 9 months, *P* < 0.001, respectively, Supplementary Figure 1B, F), while patients with *SRSF2* mutation had a significantly inferior OS (14.5 month *vs*. 29.5 months, *P* = 0.021, Supplementary Figure 1C) and a trend of shorter DFS than those without the mutation (0 month *vs*. 9 months, *P* = 0.172, Supplementary Figure 1D). As mutations of all three individual SF implicated a poor response to treatment and inferior outcome, we therefore analysed the clinical relevance of SF mutations as a whole. Patients with SF mutations had significantly poorer OS and DFS than those without SF mutation (median, 6 months *vs*. 38 months, *P* < 0.001, and median, 0 month *vs.* 10 months, *P* < 0.001, respectively, Figure 2A, 2B). The prognostic differences remained similar among the patients with non-M3 AML (median, 6 months *vs.* 25 months, *P* < 0.001 and median, 0 month *vs.* 9 months, < 0.001, respectively) and those with intermediate-risk cytogenetics (median, 7 months *vs.* 23.5 months, *P* < 0.001, Figure 2C and median, 0 month *vs.* 7.5 months, *P* < 0.001, Figure 2D, respectively). The same were also true for the subgroup of 161 patients with normal karyotype (median, 4.5 months *vs.* 61 months, *P* < 0.001, Figure 2E and median, 0 month *vs.* 10 months, *P* < 0.001, Figure 2F, respectively).

In multivariate analysis (Table 4), the independent poor risk factors for OS were older age >50 years, higher white blood cell (WBC) counts >50,000/μL, unfavorable-risk cytogenetics and mutations of SF (RR 2.243, 95% CI 1.380-3.647, *P* = 0.001), *TP53*, *RUNX1*, *WT1* and *DNMT3A*. On the other hand, *CEBPA*^double-mutation^ and *NPM1* mutation in the absence of *FLT3-*ITD (*NPM1*^+^/*FLT3-*ITD^−^) were independent favorable prognostic factors. There was a trend of better OS in patients with *IDH2* mutation (RR 0.539, 95% CI 0.284-1.020, *P* = 0.058). Similarly, the independent poor risk factors for DFS included older age > 50 years, higher WBC counts >50,000/μL, unfavorable-risk cytogenetics, and SF, *TP53*, *RUNX1*, *WT1* and *DNMT3A* mutations. On the other hand, *NPM1*^+^/*FLT3-*ITD^−^ was an independent favorable prognostic factor. In the 229 patients with intermediate-risk cytogenetics, the SF mutation was still an independent poor prognosis for OS and DFS (RR, 2.999; 95% CI, 1.002-2.999, *P* = 0.049 and RR, 1.705; 95% CI, 1.028-2.827, *P* = 0.039, respectively, Supplementary Table 5).

Intriguingly, among the 97 patients receiving allogeneic HSCT, either in first CR (*n* = 45) or beyond (*n* = 52), the poor prognostic impact of SF mutation on OS and DFS was lost (*P* = 0.439 and *P* = 0.348, respectively). It seems that HSCT may ameliorate the poor survival impact of SF mutations, similar to *RUNX1* mutations.[12, 13] However, because the number of patients who had SF mutations and received HSCT was limited in our cohort, further studies in more patients are needed to clarify this point.

**Figure 3:**
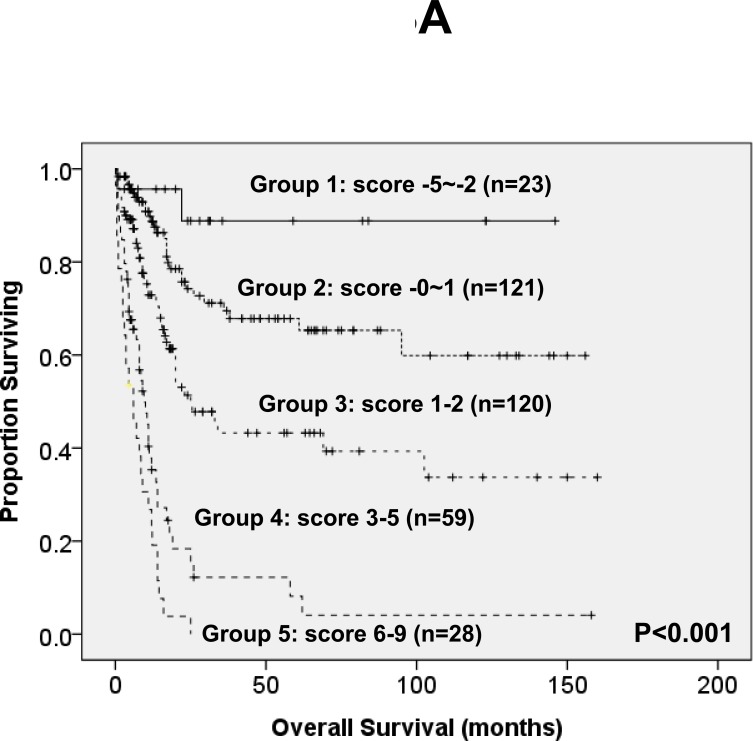

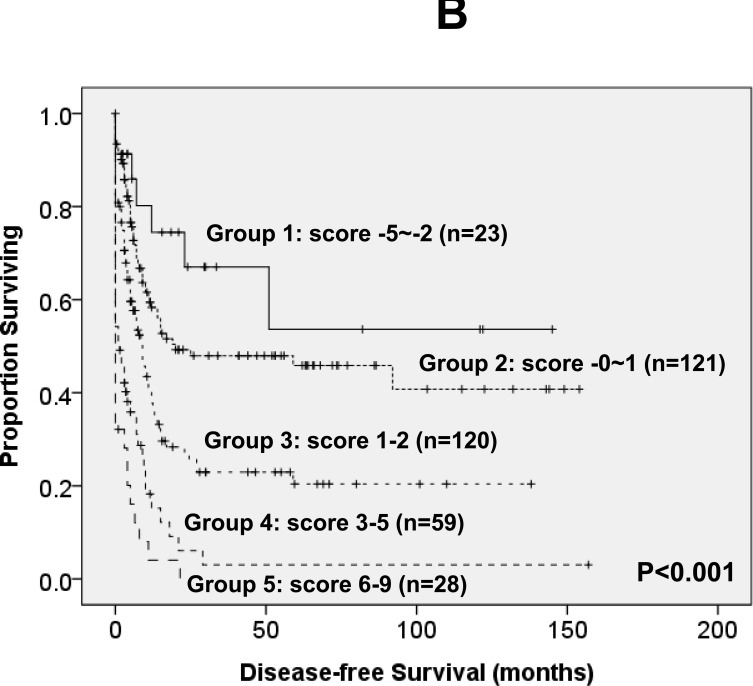
Kaplan-Meier survival curves for overall survival (A) and disease-free survival (B) in AML patients based on scoring system (*P* < 0.001 for both OS and DFS) AML patients were grouped according to scoring system based on SF mutation and 10 other prognostic markers (*CEBPA*^double-mutation^, *NPM1*/*FLT3-*ITD, *IDH2*, *TP53, WT1*, *RUNX1* and *DNMT3A* mutations, cytogenetics, age and WBC counts at diagnosis). A score of -3 was assigned for *NPM1*^+^/*FLT3-*ITD^−^ and -2 for *CEBPA*^double-mutation^ and *IDH2* mutation whereas a score of +3 for *TP53* mutation and +2 for other factors associated with an adverse outcome (SF, *DNMT3A*, *WT1* and *RUNX1* mutations, older age, higher WBC counts at diagnosis and unfavorable cytogenetics). The algebraic summation of these scores of each patient was the final score. This score system divided the AML patients into five groups with different clinical outcomes (*P* < 0.001 for both OS and DFS). The 12 patients without chromosome data were not included in the analysis.

To better stratify the AML patients into different risk groups, a scoring system incorporating SF mutations with ten other prognostic factors, including age, WBC counts, cytogenetics at diagnosis, *NPM1*/*FLT3-*ITD, and mutations of *CEBPA*, *IDH2, TP53, DNMT3A*, *RUNX1* and *WT1*, into survival analysis was formulated based on the results of our Cox proportional hazards model. The weight of the each variable was based on the value of relative risk (Table 4). To simplify the clinical utilization, a score of -3 was assigned for *NPM1*^+^/*FLT3-*ITD^−^ and -2 for *CEBPA*^double-mutation^ and *IDH2* mutation whereas a score of +3 for *TP53* mutation and +2 for other factors associated with an adverse outcome (SF, *DNMT3A*, *WT1* and *RUNX1* mutations, older age, higher WBC counts at diagnosis and unfavorable cytogenetics). The algebraic summation of these scores of each patient was the final score. This score system divided the AML patients into five groups with different clinical outcomes (*P* < 0.001 for both OS and DFS, Figure 3).

**Table 4:** Multivariate Analysis (Cox regression) on the Overall Survival and Disease-free Survival

Variables	Overall Survival				Disease-free Survival			
		95% CI				95% CI		
	RR	Lower	Upper	*P*	RR	Lower	Upper	*P*
Age^†^	2.228	1.598	3.106	<0.001*	1.344	1.016	1.779	0.038*
WBC^§^	2.192	1.539	3.123	<0.001*	1.731	1.285	2.331	<0.001*
Karyotype^Ψ^	2.227	1.230	4.032	0.008*	1.792	1.087	2.955	0.022*
*NPM1/FLT3-ITD*^ζ^	0.343	0.171	0.686	0.002*	0.304	0.163	0.567	<0.001*
*CEBPA*^‡^	0.462	0.238	0.896	0.022*	0.630	0.392	1.014	0.057
*RUNX1*	1.942	1.129	3.339	0.016*	1.788	1.138	2.809	0.012*
*WT1*	2.560	1.508	4.346	<0.001*	2.469	1.614	3.778	<0.001*
*ASXL1*	1.126	0.622	2.039	0.695	0.978	0.562	1.704	0.938
IDH2**	0.539	0.284	1.020	0.058	0.840	0.530	1.333	0.459
*DNMT3A*	1.919	1.166	3.158	0.010*	2.130	1.400	3.241	<0.001*
*TP53*	3.613	1.598	8.167	0.002*	2.824	1.372	5.812	0.005*
SF	2.243	1.380	3.647	0.001*	2.136	1.376	3.314	0.001*

Abbreviation: RR, relative risk; CI, confidence interval, SF, splicing factor.

* Statistically significant (P < 0.05)

† Age > 50 relative to Age ≤50 (the reference)

§ WBC greater than 50,000/μL *vs.* 50,000/μL or less

ζ *NPM1*^mut^/*FLT3-ITD*^neg^ *vs.* other subtypes

‡ *CEBPA*^double-mutation^ *vs.* others

Ψ unfavorable cytogenetics *vs.* others

** *IDH2* mutations included R140 and R172 mutations

### Sequential studies of SF mutations

SF mutations were serially studied in 489 samples from 163 patients, including 11 patients with SF mutations and 152 patients without the mutation at diagnosis (Table 5). Among the nine patients with SF mutations who obtained a CR and had available samples for study, eight lost the original mutation at remission status, but one (patient 22) retained it (Table 5). In addition to *U2AF1* mutation, patient 22 also harbored concurrent mutations of *NRAS* and *IDH2* at diagnosis and these two mutations disappeared at CR. The amplitude of the mutant sequence of *U2AF1* in this patient was much lower at CR compared to that at diagnosis and relapse (Supplementary Figure 2). It implied that the cells with the mutation were present as a minor population at remission suggesting that the mutation was not hereditary, but acquired, and such residual leukemia cells would then cause relapse.

In the seven patients who had available samples for serial study at relapse, the original SF mutation could be detected at relapse in six patients (patients 3, 8, 14, 22, 26 and 34), but was lost in one (*SRSF2* mutant in patient 36). Because direct sequencing might not be sensitive enough to detect low level of SF mutation signal, we therefore sequenced TA clones of the PCR product from patient 36 at relapse. The original *SRSF2* mutant could be detected in one out of 45 clones. Interestingly, acquisition of novel mutations was noted at relapse in three SF*-*mutated patients (patients 3, 8 and 14, Table 5). On the other side, among the 152 patients who had no SF mutation at diagnosis, none acquired SF mutation at relapse.

**Table 5:** Sequential studies in the AML patients with SF mutations*

UPN	Interval^†^ (months)	Status	karyotype	SF mutation	Other mutations
3		Initial	46,XX	*SF3B1* (K666N)	*RUNX1, TET2, P53*
	4	CR	ND	—	
	7.5	Relapse	ND	*SF3B1* (K666N)	*RUNX1, TET2, P53, FLT3*/ITD
8		Initial	46,XY,t(15;17)(q22;q21)	*SF3B1* (K666N)	—
	12	Relapse	46,XY,t(15;17)(q22;q21)	*SF3B1* (K666N)	*ASXL1*
14		Initial	45,XY,-7	*U2AF1* (Q157R)	*FLT3*/TKD, *ASXL1* (*P1377SfsX3*)
	4	CR	46,XY	—	—
	16.5	Relapse	ND	*U2AF1* (Q157R)	*ASXL1* (*S1255X*)
15		Initial	46,XY	*U2AF1* (S34Y)	*PTPN11, ASXL1, DNMT3A*
	5.4	CR	ND	—	—
22		Initial	47,XY,+8	*U2AF1* (E159_M160insYE)	*NRAS, IDH2*
	4.2	CR1	46,XY	*U2AF1* (E159_M160insYE)	—
	11	Relapse 1	48,XY,+8,+15	*U2AF1* (E159_M160insYE)	*NRAS, IDH2*
	2	CR2	ND	*U2AF1* (E159_M160insYE)	—
	8	Relapse 2	46-48,XY,+X,+15	*U2AF1* (E159_M160insYE)	*NRAS, IDH2*
26		Initial	47,XY,+11	*U2AF1* (S34Y)	*FLT3*/ITD, *MLL*/PTD
	8.7	Relapse	ND	*U2AF1* (S34Y)	*FLT3*/ITD, *MLL*/PTD
34		Initial	46,XY,del(7)(q22q36)	*SRSF2* (P95H)	*NPM1, RUNX1, ASXL1*
	5.5	CR1	46,XY	—	*ASXL1*
	4	Relapse 1	46,XY	*SRSF2* (P95H)	*NPM1, RUNX1, ASXL1*
36		Initial	48,XY,+add(1)(p13),+8	*SRSF2* (P95R)	*IDH1*
	1	CR1	46,XY	—	—
	7.5	Relapse 1	46,XY	*SRSF2* (P95R)^††^	*IDH1*
37		Initial	46,XY	*SRSF2* (P95L)	*CEBPA, IDH2*
	2.5	CR1	ND	—	—
47		Initial	47,XX,+8	*SRSF2* (P95H)	*CEBPA, IDH2, DNMT3A*
	2	CR1	46,XX	—	—
54		Initial	46,XY	*SRSF2* (P95_R102del)	*RUNX1, IDH2*
	5	CR1	ND	—	—

Abbreviations: UPN, unique patient number; CR, complete remission; ND, not done.

* The data of serial studies in other 152 patients, who did not have *SF* mutation both at diagnosis and relapse were not shown in this table

† Interval between the two successive status

†† The *SRSF2* (patient 36) mutation could be detected by TA cloning (one out of 45 clones), but not by direct sequencing, at relapse.

## DISCUSSION

Most studies on SF mutations in AML were focused on small patients cohorts.[2, 8, 9, 11] To the best of our knowledge, this study recruited the largest cohort of *de novo* AML. Patients with antecedent hematological diseases, family history of myeloid neoplasms or therapy-related AML were excluded the same way we did previously.[14, 15] We found that SF mutation was associated with distinct clinic-biological features and was a poor prognostic factor in AML patients, independent of age, WBC counts, karyotype and other genetic markers.

Mutations of the SF genes were identified in 54 (10.8%) patients, most commonly in those with intermediate-risk cytogenetics (13.2%). Similar to the data in MDS, the majority of mutations occurred in hotspot areas: K666N and K700E in *SF3B1*, S34 and Q157 in *U2AF1* and P95 in *SRSF2*. The incidence of SF mutations in AML varied from 4.5%-12.5% in different reports.[2, 8-11] Yoshida et al found *SF3B1, U2AF1* and *SRSF2* mutations in 2.6%, 1.3% and 0.7%, respectively, of 151 AML patients.[2] Kihara et al reported 4.5% of 197 patients harbored SF mutations, including *SF3B1* (1.5%), *U2AF1* (1.5%), *SRSF2* (1%) and *ZRSR2* (0.5%) mutations. By analyzing the mutations in eight hotspots of SF genes in 325 patients, Taskesen et al showed 1.8% of AML patients had mutations in *SF3B1*, 1.2% in *U2AF1* and 4.6% in *SRSF2*.[10] In a cohort of 200 adult AML patients reported by the Cancer Genome Atlas (TCGA), the incidence of mutations in 21 spliceosome genes detected by either whole-genome sequencing or whole-exome sequencing was 12.5%; among them, *SF3B1* mutation was found in 0.5%, *U2AF1* mutation in 4% and *SRSF2* mutation in 0.5%.[11] Surprisingly, mutations in *SRSF2* gene occurred in 81% of AML patients with isolated trisomy 13.[16] The reason of the variability in the incidence of SF mutations in different studies is unknown but may be due to differences in ethnic background, patient population selected (age range, FAB subtypes and karyotype, etc), the regions of SF genes screened, and the methods used. We analyzed exons 14-15 in *SF3B1* genes, exons 2 and 6 in *U2AF1* genes and exon 2 in *SRSF2* gene to avoid missing some mutations outside hotspot regions. A higher frequency of *SRSF2* mutations in this study might be partially due to age effect; elder patients were also enrolled in this cohort and *SRSF2* mutation is closely associated with older age in myeloid neoplasm.[17]

Although a close association was observed between SF mutations and mutations in certain genes, especially those related to epigenetic modifications, in MDS (such as *SF3B1* mutation with *DNMT3A* mutation, *SRSF2* mutation with mutations of *RUNX1, IDH* and *ASXL1* genes and *U2AF1* mutation with mutations of *ASXL1* and *DNMT3A*),[4, 6, 18, 19], little is known about the interaction between SF mutations and other molecular genetic alterations in AML patients. In a study of mutational status of three SF genes (*SF3B1, U2AF1* and *SRSF2*), *NPM1, FLT3, CEBPA, IDH1, DNMT3A, ASXL1* and *NRAS/KRAS* in 344 patients, including 47 refractory anemia with excess blasts (RAEB), 29 AML with low BM blast count and other AML patients, Taskesen et al could not find any molecular association.[10] However, with the help of combined genome-wide mRNA expression and DNA-methylation profiling they identified two distinct patient clusters highly enriched for SF-mutated RAEB/AML. One cluster was associated with erythroid phenotype; the other was correlated with *NRAS/KRAS* mutation (10 out of 25 patients, 40%). However, the reason why these two clusters were defined only by combined genome-wide mRNA expression and DNA-methylation profiling was unclear. In this study, we found SF mutations rarely occurred alone; 49 (90.7%) of 54 patients with SF mutations showed additional molecular abnormalities at diagnosis. This finding is in agreement with the concept that the development of AML requires concerted cooperation of different molecular genetic alterations.[11, 20] Intriguingly, patients with SF mutations had significantly higher incidences of *RUNX1, ASXL1, IDH2* and *TET2* mutations than those without the mutation, similar to the findings in MDS.[4, 6, 18, 19]

To the best of our knowledge, this study is the first to evaluate the dynamic change of SF mutation during disease progression in a large cohort of patients with *de novo* AML. In contrast to the instability of *FLT3*-ITD during disease evolution,[21] we found that the SF mutation seemed rather stable, analogous to *DNMT3A* mutations[14, 22] At relapse, the original SF mutations in all seven SF-mutated patients studied were retained, but the mutant level in one of them was much reduced at the time of AML relapse as it could only be detected by a sensitive cloning technique, but not by direct sequencing. (patient 36, Table 5) On the other side, among the 152 patients who had no SF mutation at diagnosis, all remained germline of the genes during clinical follow-ups. Taken together, these findings suggest that SF mutations were quite stable during disease evolution and may play an important role in development, but not progression of AML.

Few studies regarding the prognostic relevance of SF mutations in *de novo* AML have been reported. In a study of Taskesen et al, only one distinct SF-mutant patient cluster enriched for *NRAS/KRAS* mutation (cluster 3, 7.3% of 344 patients) had poorer prognosis. Patients with isolated trisomy 13 reported by Herold et al, in whom high frequencies of mutations in *SRSF2* (81%) and *RUNX1* (75%) were noted, had a dismal outcome.[16] In this study, we distinctly identified that SF mutation was an important prognostic factor, independent from all other variables in both total cohort and patients with intermediate-risk cytogenetics. Although *SF3B1* mutations have been shown to predict better OS in MDS patients,[3, 19, 23, 24] we found the mutation was associated with a lower CR rate (Supplementary Table 2A) and shorter survival in *de novo* AML patients (Supplementary Figure 1A, B). The reason why *SF3B1* mutation has different impact on clinical outcome between patients with MDS and AML remains to be explored. In fact, the reports concerning the prognostic impact of *SF3B1* mutation in MDS showed inconsistent and conflicting results.[3, 19, 23, 24] The good prognostic impact of *SF3B1* mutation could not be demonstrated in MDS patients in some studies.[3, 19, 23, 24] It was suggested the close association of *SF3B1* mutation with old age and *DNMT3A* mutation and different treatment regimens might influence the implication of this mutation on survival of MDS patients.[19, 24] In AML, Lindsley et al[25] first showed that SF mutations as well as *ASXL1, EZH2, BCOR*, and *STAG2* mutations were highly specific for secondary AML, and were secondary-type mutations in therapy-related AML and elderly *de novo* AML that defined a distinct subgroup of patients with poor outcome. In this study, we only recruited *de novo* AML patients, the same cohort as we reported previously.[14, 15] Secondary AML patients were carefully excluded and SF mutations in this study were closely associated with intermediate-risk cytogenetics, but not poor-risk cytogenetics or complex karyotype, which is frequently seen in secondary AML. The findings from this study reflected the poor prognostic implication of SF mutations in *de novo* AML patients.

Intriguingly, the poor prognostic impact of SF mutation in OS and DFS was lost if the patients received allogeneic HSCT. In other words, HSCT may ameliorate the poor survival impact of SF mutations. Further studies in more patients are needed to clarify this point. To better stratify AML patients into different risk groups, a survival scoring system incorporating SF mutation and ten other prognostic factors, including age, WBC counts, cytogenetics, *NPM1/FLT3-ITD, CEBPA, IDH2, RUNX1, WT1, DNMT3A* and *TP53* mutations, into survival analysis was formulated. Indeed, this scoring system was more powerful than single marker to separate patients into different prognostic groups. Further studies in independent cohorts are needed to validate the clinical implication of the proposed scoring system.

There was one potential flaw and limitation in this study. We did not analyze the mutations of all 21 spliceosome genes; the results we obtained might only reflect the clinical relevance of mutations in the three SF genes we analyzed. However, *SF3B1, U2AF1* and *SRSF2* mutations are the most frequent SF mutations in myeloid neoplasms and can be easily detected by Sanger's sequencing.[2, 11] The finding that mutations in these three SF genes predict poor prognosis suggests routine test of these mutations may be helpful in the clinical management of AML patients.

In summary, this study demonstrated that SF-mutated patients had specific clinic-biologic features and cytogenetic changes. SF mutations were closely associated with *RUNX1, ASXL1, IDH2* and *TET2* mutations. Furthermore, the SF mutation was an independent poor-risk factor for OS and DFS among total cohort and patients with intermediate-risk cytogenetics. Incorporation of SF mutation with ten other prognostic factors into survival analyses can better stratify AML patients into different risk groups. Sequential study during the clinical course showed that SF mutations were quite stable during AML evolution. These mutations can be potential targets for novel therapies and biomarkers for disease monitoring.

## MATERIALS AND METHODS

### Subjects

From March 1995 to Dec 2008, a total of 500 adult patients with newly diagnosed *de novo* AML according to the French-American-British (FAB) criteria at the National Taiwan University Hospital (NTUH) were enrolled as previously described.[14, 15] Patients with antecedent hematological diseases, history of cytopenia, family history of myeloid neoplasms or therapy-related AML were excluded. Among them, 363 (72.6%) patients received standard induction chemotherapy (Idarubicin 12 mg/m^2^ per day on days 1-3 and Cytarabine 100 mg/m^2^ per day on days 1-7) and then consolidation chemotherapy with 2-4 courses of high-dose Cytarabine (2000 mg/m^2^ q12h days 1-4, total 8 doses), with or without an anthracycline (Idarubicin or Novatrone), after achieving complete remission (CR).[14, 15] The patients with acute promyelocytic leukemia (M3 subtype) received concurrent all-trans retinoic acid and chemotherapy. The remaining 137 patients received palliative therapy due to underlying comorbidity or based on the decision of the patients. Forty-five patients received allogeneic hematopoietic stem cell transplantation (HSCT) in first CR and 52 in relapse/refractory status or second CR or beyond. This study was approved by the Institutional Review Board of the NTUH; and written informed consent was obtained from all participants in accordance with the Declaration of Helsinki.

### Cytogenetics

Chromosomal analyses were performed as described previously.[26]

### Immunophenotype analysis

A panel of monoclonal antibodies to myeloid associated antigens, including CD13, CD33, CD11b, CD15, CD14, and CD41a, as well as lymphoid-associated antigens, including CD2, CD5, CD7, CD19, CD10, and CD20, and lineage nonspecific antigens HLA-DR, CD34, and CD56 were used to characterize the phenotypes of the leukemia cells as previously described.[14]

### Mutation analysis

Mutation analysis of SF genes, including *SF3B1, SRSF2* and *U2AF1*, was performed by polymerase chain reaction (PCR) and direct sequencing.[17-19] Abnormal sequencing results were confirmed by at least two repeated analyses. Sequential analysis of SF mutations during the clinical course was performed in 489 samples from 163 patients. Mutation analyses of 18 other relevant molecular marker genes, including Class I mutations, such as *FLT3*/ITD and *FLT3*/TKD,[27] *NRAS*,[28] *KRAS*,[28] *JAK2*,[28] *KIT*[29] and *PTPN11*[29] mutations and Class II mutations, such as *CEBPA*[30] and *RUNX1*[13] mutations, as well as *NPM1*,[31] *WT1*,[32] *TP53*[33] and those genes related to epigenetic modification, such as *MLL*/PTD,[34] *ASXL1*,[35] *IDH1*,[36] *IDH2*,[37] *TET2*[38] and *DNMT3A*[14] mutations were performed as previously described. To detect SF mutations at diagnosis, we used DNA amplified *in vitro* from patients’ BM cells by Illustra^TM^ GenomiPhi V2 DNA amplification kit as described by the manufacturer (GE Healthcare, Buckinghamshire, UK). All the mutations detected in such samples were verified in the original non-amplified samples.

### TA cloning analysis

For the patients with discrepancy of the mutation status of the SF genes in paired samples, Taq polymerase-amplified (TA) cloning was performed in the samples without detectable mutant by direct sequencing as previously described.[28]

### Statistical analysis

The discrete variables of patients with and without SF mutation were compared using the chi-square tests, but if the expected values of contingency tables were smaller than 5, Fisher exact test was used. If the continuous data were not normally-distributed, Mann-Whitney U tests were used to compare continuous variables and medians of distributions. To evaluate the impact of SF mutation on clinical outcome, only the patients who received conventional standard chemotherapy, as mentioned above, were included in analysis.[14, 15] OS was measured from the date of first diagnosis to the date of last follow-up or death from any cause, whereas relapse was defined as a reappearance of at least 5% leukemic blasts in a BM aspirate or new extramedullary leukemia in patients with a previously documented CR.[39] Disease-free (DF) status indicated that the patient achieved CR and did not relapse by the end of this study. Cox regression survival estimation was used to plot survival curves and to test the difference between groups. Multivariate Cox proportional hazard regression analysis was used to investigate independent prognostic factors for OS and DFS. The proportional hazards assumption (constant hazards assumption) was examined by using Time-Dependent Covariate Cox regression before conducting multivariate Cox proportional hazard regression. The variables including age, WBC counts, karyotype, *NPM1/FLT3-ITD, CEBPA, IDH2, WT1, RUNX1, ASXL1, DNMT3A* and *TP53* mutations were used as covariates. Those patients who received HSCT were censored at the time of HSCT in survival analysis to ameliorate the influence of the treatment.[14, 15] A *P*-value < 0.05 was considered statistically significant. All statistical analyses were performed with the SPSS 19 (SPSS Inc., Chicago, IL, USA) and Statsdirect (Cheshire, England, UK).

## SUPPLEMENTARY MATERIAL TABLES AND FIGURES


