# Supplementary material for: Splicing factor mutations predict poor prognosis in patients with de novo acute myeloid leukemia
Source: Oncotarget. 2016 Jan 24;7(8):9084–101. doi: 10.18632/oncotarget.7000 (PMC4891028; doi:10.18632/oncotarget.7000)

Splicing factor mutations predict poor prognosis in patients with *de novo* acute myeloid leukemia

Supplementary Material

Supplementary Table 1
Comparison of immunophenotypes of leukemia cells between AML patients with and without SF mutation

Antigens	Total patients examined	Percentage of patients with the antigen expression			P
		Whole cohort	SF-Mutated patients	SF-Wild patients	
HLA-DR	476	70	84.6	68.2	0.0156
CD13	478	94.6	98.1	94.1	0.3407
CD33	477	91	82.7	92	0.0379
CD11b	149	29.8	28.6	29.6	>0.9999
CD14	466	13.5	12.2	13.8	>0.9999
CD19	469	7.0	12.2	7.2	>0.9999
CD10	414	0.5	5.8	0.5	>0.9999
CD7	479	20.0	11.5	20.5	0.1404
CD2	473	4.2	1.9	4.5	0.7122
CD15	473	44.8	34.6	46.1	0.1396
CD34	473	65.1	80.8	63.2	0.0131
CD56	428	23.8	12.2	25.3	0.0493

Supplementary Table 2

Supplementary Table 2A
Comparison of clinical and laboratory features between AML patients with and without *SF3B1* mutation

Variables	Total (n=500)	*SF3B1*-Mutated (n=12, 2.4%)	*SF3B1*-Wild (n=488, 96.4%)	P value
**Sex**†				>0.9999
Male	285	7 (2.5)	278 (97.5)	
Female	215	5 (2.3)	210 (97.7)	
**Age (year)**‡	51 (15-90)	68.5 (31-86)	51 (15-90)	0.0123
**Lab data**‡				
WBC (/μL)	19075 (120-627800)	36870 (7300-627800)	17710 (120-423000)	0.081
Hb (g/dL)	8 (2.9-16.2)	7.5 (4.4-11.2)	8(2.9-16.2)	0.5479
Platelet (×1,000 /μL)	42 (2-802)	89.5 (15-331)	41(2-802)	0.0178
Blast (/μL)	7401 (0-456725)	14831(1404-456725)	7243 (0-369070)	0.1265
LDH (U/L)	889 (206-15000)	1248 (450-4220)	840(206-15000)	0.17
**FAB**†				
M0	10	0 (0)	10 (100)	>0.9999
M1	112	6 (5.4)	106 (94.6)	>0.9999
M2	171	2 (1.2)	169 (98.8)	0.235
M3	38	1 (2.6)	37 (97.4)	>0.9999
M4	124	2 (1.6)	122 (98.4)	0.7388
M5	24	1 (4.2)	23 (95.8)	0.4496
M6	12	0 (0)	12 (100)	>0.9999
Undetermined	9	0 (0)	9 (100)	>0.9999
**Induction response***	363	9	354	
CR	284	2 (22.2)	282 (79.7)	0.0005
PR/Refractory	54	2 (22.2)	50 (14.1)	0.6223
Induction death	25	3 (33.3)	22 (6.2)	0.0185
**Relapse***	144	2 (100)	142 (50.4)	0.4983

†number of patients (%)

‡ median (range)

*only the 363 patients, including 9 with *SF3B1*mutation and 354 without, who received conventional intensive induction chemotherapy and then consolidation chemotherapy if CR was achieved, as mentioned in the text, were included in the analysis.

Supplementary Table 2B
Comparison of clinical and laboratory features between AML patients with and without *SRSF2* mutation

Variables	Total (n=500)	*SRSF2*-Mutated (n=27, 3.6%)	*SRSF2*-Wild (n=473, 94.6%)	P value
**Sex**†				0.0087
Male	285	22 (7.7)	263 (92.3)	
Female	215	5 (2.3)	210 (97.7)	
**Age (year)**‡	51 (15-90)	70 (42-90)	50(15-90)	<0.0001
**Lab data**‡				
WBC (/μL)	19075 (120-627800)	13920 (890-253390)	19440(120-627800)	0.9338
Hb (g/dL)	8 (2.9-16.2)	9.4(3.8-16.2)	7.9 (2.9-14)	0.0085
Platelet (×1,000 /μL)	42 (2-802)	32 (7-455)	42(2-802)	0.4495
Blast (/μL)	7401 (0-456725)	4326 (103-212772)	7472(0-456725)	0.9344
LDH (U/L)	889 (206-15000)	859 (329-7930)	852(206-15000)	0.9051
**FAB**†				
M0	10	1 (10)	9 (90)	0.429
M1	112	5 (4.5)	107 (95.5)	0.813
M2	171	6 (3.5)	165 (96.5)	0.214
M3	38	0 (0)	38 (100)	0.2513
M4	124	9 (7.3)	115 (92.7)	0.3579
M5	24	3 (12.5)	21 (87.5)	0.133
M6	12	0 (0)	12 (100)	>0.9999
Undetermined	9	3 (33.3)	6 (66.7)	0.0095
**Induction response***	363	11	352	
CR	284	5 (45.5)	279 (79.3)	0.0162
PR/Refractory	54	3 (27.3)	51 (14.5)	0.2152
Induction death	25	3 (27.3)	22 (6.3)	0.033
**Relapse***	144	2 (40)	142 (50.9)	0.6809

†number of patients (%)

‡ median (range)

*only the 363 patients, including 11 with *SRSF2* mutation and 352 without, who received conventional intensive induction chemotherapy and then consolidation chemotherapy if CR was achieved, as mentioned in the text, were included in the analysis.

Supplementary Table 2C
Comparison of clinical and laboratory features between AML patients with and without *U2AF1* mutation

Variables	Total (n=500)	*U2AF1*-Mutated (n=15, 3.0%)	*U2AF1*-Wild (n=485, 97.0%)	P value
**Sex**†				0.1093
Male	285	12 (4.2)	273 (95.8)	
Female	215	3 (1.4)	212 (98.6)	
**Age (year)**‡	51 (15-90)	48 (22-75)	51 (15-90)	0.6777
**Lab data**‡				
WBC (/μL)	19075 (120-627800)	6740 (120-144900)	20270(300-627800)	0.1355
Hb (g/dL)	8 (2.9-16.2)	6.3 (3.7-13.1)	8(2.9-16.2)	0.0707
Platelet (×1,000 /μL)	42 (2-802)	25 (6-277)	42.5(2-802)	0.0531
Blast (/μL)	7401 (0-456725)	1960 (14-136206)	7560(0-456725)	0.1698
LDH (U/L)	889 (206-15000)	654 (288-2650)	860(206-15000)	0.0924
**FAB**†				
M0	10	2 (20)	8 (80)	0.0329
M1	112	3 (2.7)	109 (97.3)	>0.9999
M2	171	4 (2.3)	167 (97.7)	0.783
M3	38	0 (0)	38 (100)	0.618
M4	124	5 (4.0)	119 (96.0)	0.5426
M5	24	0 (0)	24 (100)	>0.9999
M6	12	1 (8.3)	11 (91.7)	0.309
Undetermined	9	0 (0)	9 (100)	>0.9999
**Induction response***	363	12	351	
CR	284	4 (33.3)	280 (79.8)	0.0009
PR/Refractory	54	8 (66.7)	46 (13.1)	<0.0001
Induction death	25	0 (0)	25 (7.1)	>0.9999
**Relapse***	144	3 (75)	141 (50.4)	0.6225

†number of patients (%)

‡median (range)

*only the 363 patients, including 12 with *U2AF1* mutation and 351 without, who received conventional intensive induction chemotherapy and then consolidation chemotherapy if CR was achieved, as mentioned in the text, were included in the analysis.

Supplementary Table 3
Association of SF mutations with chromosomal abnormalities*

Variables	Total	SF-Mutated	SF-Wild	P value
**Karyotype†**				
Favorable	99	1 (1.0)	98 (99.0)	<0.0001
Intermediate	318	42 (13.2)	276 (86.8)	0.008
Unfavorable	65	8 (12.3)	57 (87.7)	0.6638
Unknown	18	3 (16.7)	15 (83.3)	0.4231
Normal	223	27 (12.1)	196 (87.9)	0.3732
Simple	208	20 (9.6)	188 (90.4)	0.6542
Complex	51	4 (7.8)	47 (92.2)	0.6345
t(8;21)	42	0 (0)	42 (100)	0.0149
t(15;17)	38	1 (2.6)	37 (97.4)	0.1626
inv(16)	19	0 (0)	19 (100)	0.2446
t(11q23)	16	1 (6.3)	15 (93.7)	>0.9999
t(7;11)	10	0 (0)	10 (100)	0.6094
-5/5q-‡	2	0 (0)	2 (100)	>0.9999
-7/7q-‡	10	3 (30.0)	7 (70.0)	0.0785
+8‡	27	5 (18.5)	22 (81.5)	0.1895
+11‡	3	1 (33.3)	2 (66.7)	0.2856
+13‡	1	1 (100)	0 (0)	0.1058
+21‡	9	0 (0)	9 (100)	0.6069

*Four hundred and eighty-two patients, including 51SF-mutated and 431SF-wild patients, had chromosome data at diagnosis.

†Favorable, t(15;17), t(8;21), inv (16) ; unfavorable, -7, del(7q), -5, del(5q), 3q abnormality, complex abnormalities; Intermediate, normal karyotype and other abnormalities.

‡Only including simple chromosomal abnormalities with 2 or less changes, but not those with complex abnormalities with 3 or more aberrations.

Supplementary Table 4

Supplementary Table 4A
Association of *SF3B1* mutation with other gene mutations

Variables	No. of patients with alteration (%)			P value
	Whole cohort (n=500)	*SF3B1*-mutated patients (n=12)	*SF3B1*-wild patients (n=488)	
FLT3/ITD	113 (22.6)	4 (33.3)	109 (22.3)	0.4817
FLT3/TKD	38 (7.6)	0 (0)	38 (7.9)	0.6127
NRAS	61 (12.2)	1 (8.3)	60 (12.3)	>0.9999
KRAS	16 (3.2)	0 (0)	16 (3.3)	>0.9999
PTPN11	18 (3.6)	0 (0)	18 (3.7)	>0.9999
KIT	15 (3.0)	0 (0)	15 (3.1)	>0.9999
JAK2	3 (0.6)	0 (0)	3 (0.6)	>0.9999
WT1	33 (6.6)	0 (0)	33 (6.8)	>0.9999
NPM1	103 (20.6)	2 (16.7)	101 (20.7)	>0.9999
CEBPA	66 (13.2)	2 (16.7)	64 (13.1)	0.6645
RUNX1	62 (12.4)	5 (41.7)	57 (11.7)	0.0099
MLL/PTD	27 (5.4)	2 (16.7)	25 (5.1)	0.1327
ASXL1	50 (10.0)	1 (8.3)	49 (10.0)	>0.9999
IDH1	27 (5.4)	0 (0)	27 (5.5)	>0.9999
IDH2	55 (11)	1 (8.3)	54 (11.1)	>0.9999
TET2	66 (13.2)	2 (16.7)	64 (13.1)	0.6645
DNMT3A	70 (14.0)	4 (33.3)	66 (13.5)	0.0726
TP53	35 (7.0)	2 (16.7)	33 (6.8)	0.2023
SRSF2	27 (5.4)	0 (0)	27 (5.5)	>0.9999
U2AF1	15 (3.0)	0 (0)	15 (3.1)	>0.9999

Supplementary Table 4B
Association of *SRSF2* mutation with other gene mutations

Variables	No. of patients with alteration (%)			P value
	Whole cohort (n=500)	*SRSF2*-mutated patients (n=27)	*SRSF2*-wild patients (n=473)	
FLT3/ITD	113 (22.6)	2 (7.4)	111 (23.5)	0.0581
FLT3/TKD	38 (7.6)	1 (3.7)	37 (7.8)	0.7113
NRAS	61 (12.2)	3 (11.1)	58 (12.3)	>0.9999
KRAS	16 (3.2)	1 (3.7)	15 (3.2)	0.5943
PTPN11	18 (3.6)	1 (3.7)	17 (3.6)	>0.9999
KIT	15 (3.0)	0 (0)	15 (3.2)	>0.9999
JAK2	3 (0.6)	0 (0)	3 (0.6)	>0.9999
WT1	33 (6.6)	0 (0)	33 (7.0)	0.2438
NPM1	103 (20.6)	4 (14.8)	99 (20.9)	0.6251
CEBPA	66 (13.2)	4 (14.8)	62 (13.1)	0.7702
RUNX1	62 (12.4)	11 (40.7)	51 (10.8)	0.0001
MLL/PTD	27 (5.4)	0 (0)	27 (5.7)	0.3869
ASXL1	50 (10.0)	10 (37.0)	40 (8.5)	<0.0001
IDH1	27 (5.4)	2 (7.4)	25 (5.3)	0.6505
IDH2	55 (11)	7 (25.9)	48 (10.1)	0.0204
TET2	66 (13.2)	11 (40.7)	55 (11.6)	0.0002
DNMT3A	70 (14.0)	4 (14.8)	66 (14.0)	0.7812
TP53	35 (7.0)	1 (3.7)	34 (7.2)	0.7115
U2AF1	15 (3.0)	0 (0)	15 (3.2)	>0.9999
SF3B1	12 (2.4)	0 (0)	12 (2.5)	>0.9999

Supplementary Table 4C

Association of *U2AF1* mutation with other gene mutations

Variables	No. of patients with alteration (%)			P value
	Whole cohort (n=500)	*U2AF1*-mutated patients (n=15)	*U2AF1*-wild patients (n=485)	
FLT3/ITD	113 (22.6)	1 (6.7)	112 (23.1)	0.209
FLT3/TKD	38 (7.6)	1 (6.7)	37 (7.6)	>0.9999
NRAS	61 (12.2)	3 (20.0)	58 (12.0)	0.4105
KRAS	16 (3.2)	1 (6.7)	15 (3.1)	0.3904
PTPN11	18 (3.6)	2 (13.3)	16 (3.3)	0.0974
KIT	15 (3.0)	0 (0)	15 (3.1)	>0.9999
JAK2	3 (0.6)	0 (0)	3 (0.6)	>0.9999
WT1	33 (6.6)	1 (6.7)	32 (6.6)	>0.9999
NPM1	103 (20.6)	0 (0)	103 (21.2)	0.0492
CEBPA	66 (13.2)	1 (6.7)	65 (13.4)	0.7051
RUNX1	62 (12.4)	1 (6.7)	61 (12.6)	0.7065
MLL/PTD	27 (5.4)	1 (6.7)	26 (5.4)	0.5704
ASXL1	50 (10.0)	4 (26.7)	46 (9.5)	0.0528
IDH1	27 (5.4)	1 (6.7)	26 (5.4)	0.5704
IDH2	55 (11)	3 (20.0)	52 (10.7)	0.2219
TET2	66 (13.2)	2 (13.3)	64 (13.2)	>0.9999
DNMT3A	70 (14.0)	1 (6.7)	69 (14.2)	0.7061
TP53	35 (7.0)	0 (0)	35 (7.2)	0.6143
SRSF2	27 (5.4)	0 (0)	27 (5.6)	>0.9999
SF3B1	12 (2.4)	0 (0)	12 (2.5)	>0.9999

Supplementary Table 5

Multivariate Analysis (Cox regression) on the Overall Survival and Disease-free Survival in 229 AML Patients with Intermediate-risk Cytogenetics

Variables	Overall Survival				Disease-free Survival			
		95% CI				95% CI		
	RR	Lower	Upper	P	RR	Lower	Upper	P
Age†	3.163	1.389	3.163	<0.001*	1.324	0.944	1.857	0.104
WBC§	3.631	1.594	3.631	<0.001*	1.572	1.116	2.216	0.010*
*NPM1/FLT3-ITD*ζ	0.571	0.130	0.571	0.001*	0.239	0.128	0.449	<0.001*
CEBPA‡	0.530	0.133	0.530	<0.001*	0.383	0.232	0.633	<0.001*
WT1	3.858	1.145	3.858	0.017*	1.978	1.214	3.222	0.006*
*IDH2***	0.677	0.168	0.677	0.002*	0.607	0.372	0.989	0.045*
DNMT3A	2.391	0.877	2.391	0.148	1.554	1.027	2.353	0.037*
SF	2.999	1.002	2.999	0.049*	1.705	1.028	2.827	0.039*

Abbreviation: RR, relative risk; CI, confidence interval; SF, Splicing factor.

*Statistically significant ($P < 0.05$)

†Age $>$ 50 relative to Age $\leq$50 (the reference)

§WBC greater than 50,000/μL *vs.* 50,000/μL or less

ζ*NPM1*^{mut}/*FLT3-ITD*^{neg} *vs.* other subtypes

‡*CEBPA*$^{double\text{-}mutation}$ *vs.* others

***IDH2* mutations included R140 and R172 mutations

Supplementary Figure legend

Supplementary Figure 1

Kaplan–Meier survival curves for overall survival and disease-free survival in total 363 AML patients stratified by the status of *SF3B1* mutation (A and B), *SRSF2* mutation (C and D) and *U2AF1* mutation (E and F), respectively.

Supplementary Figure 2

Sequential analyses of*U2AF1, IDH2* and *NRAS* mutations in patient 22.

The amplitude of the mutant sequence of *U2AF1* was much lower at complete remission (CR) compared to that at diagnosis and relapse. Arrows indicated the location of the mutation (*U2AF1,* E159_M160insYE; *IDH2, R140Q* and *NRAS, Q61H*)

Supplementary Figure1

Supplementary Figure 1A

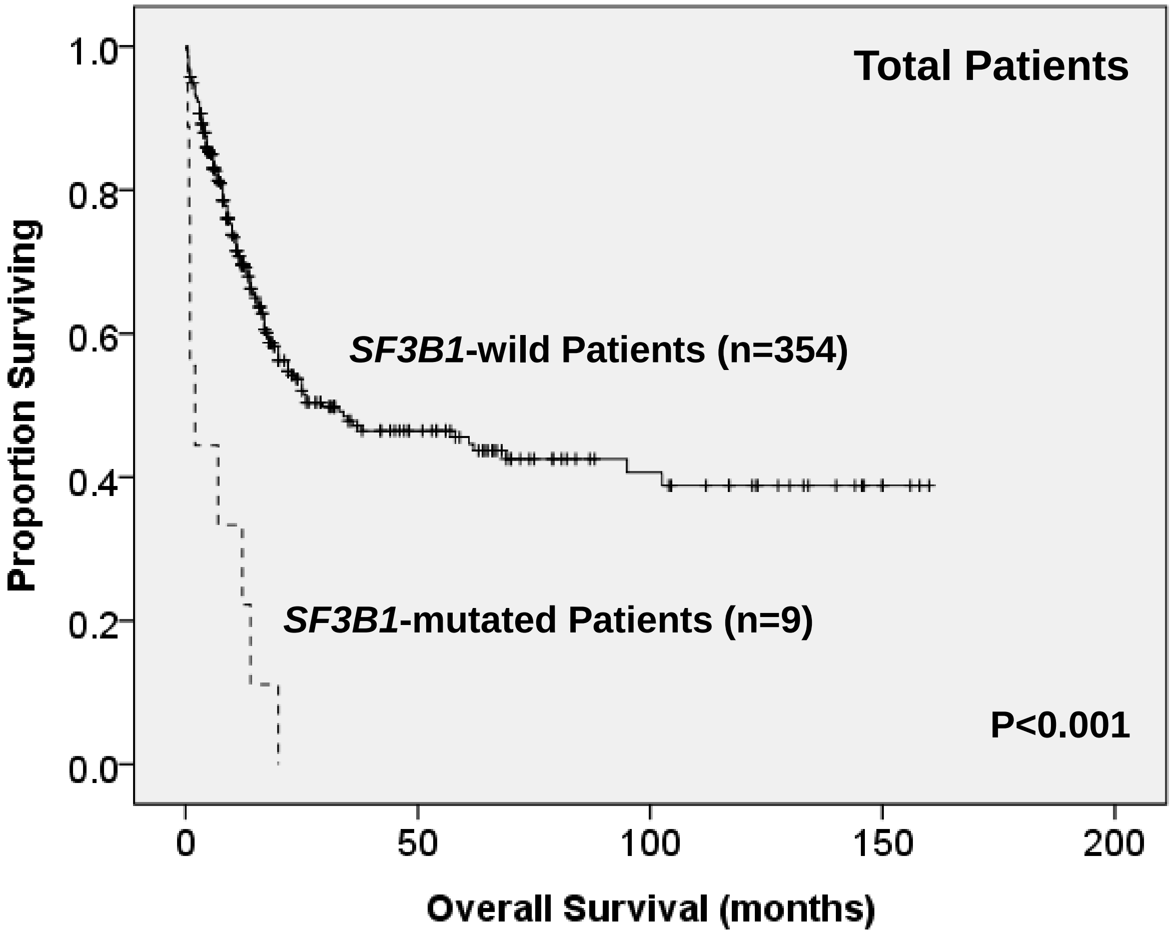

Supplementary Figure 1B

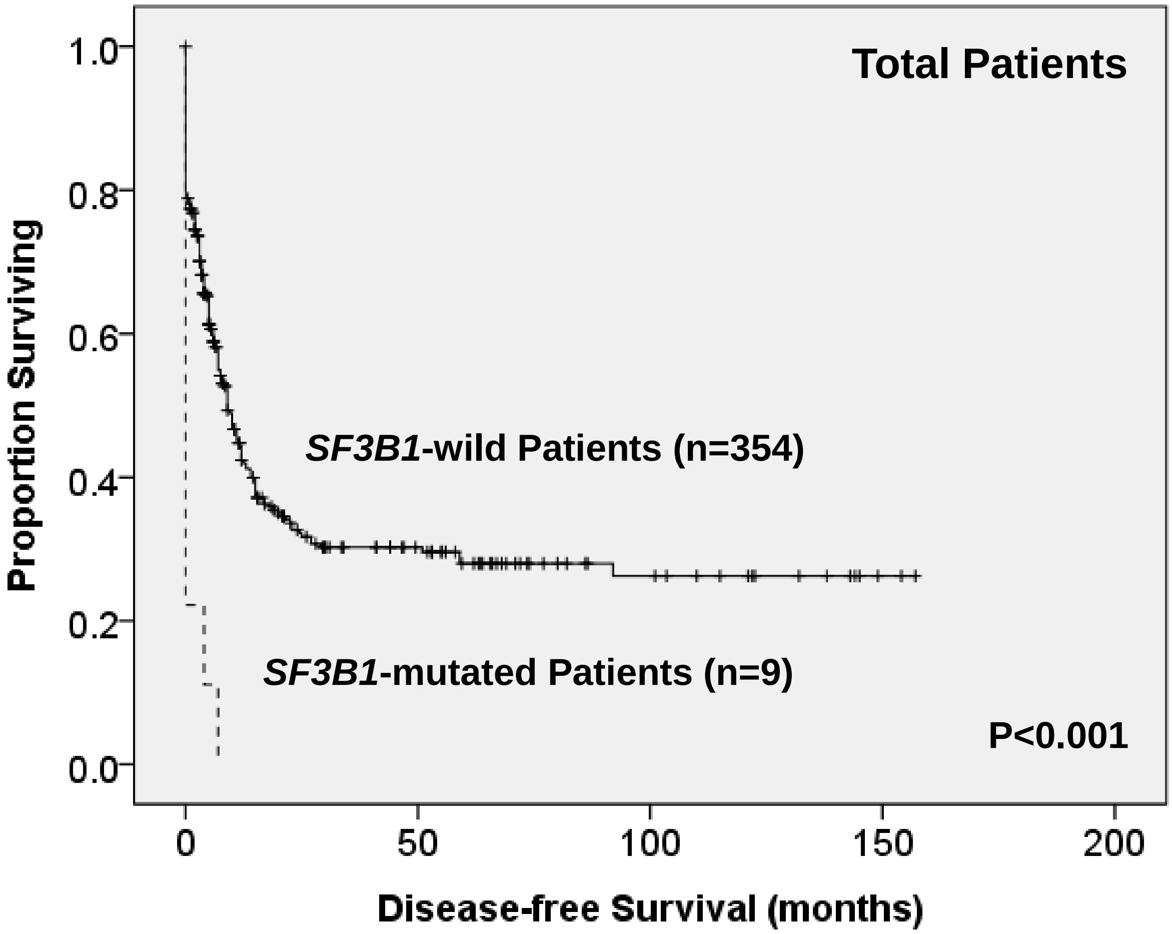

Supplementary Figure 1C

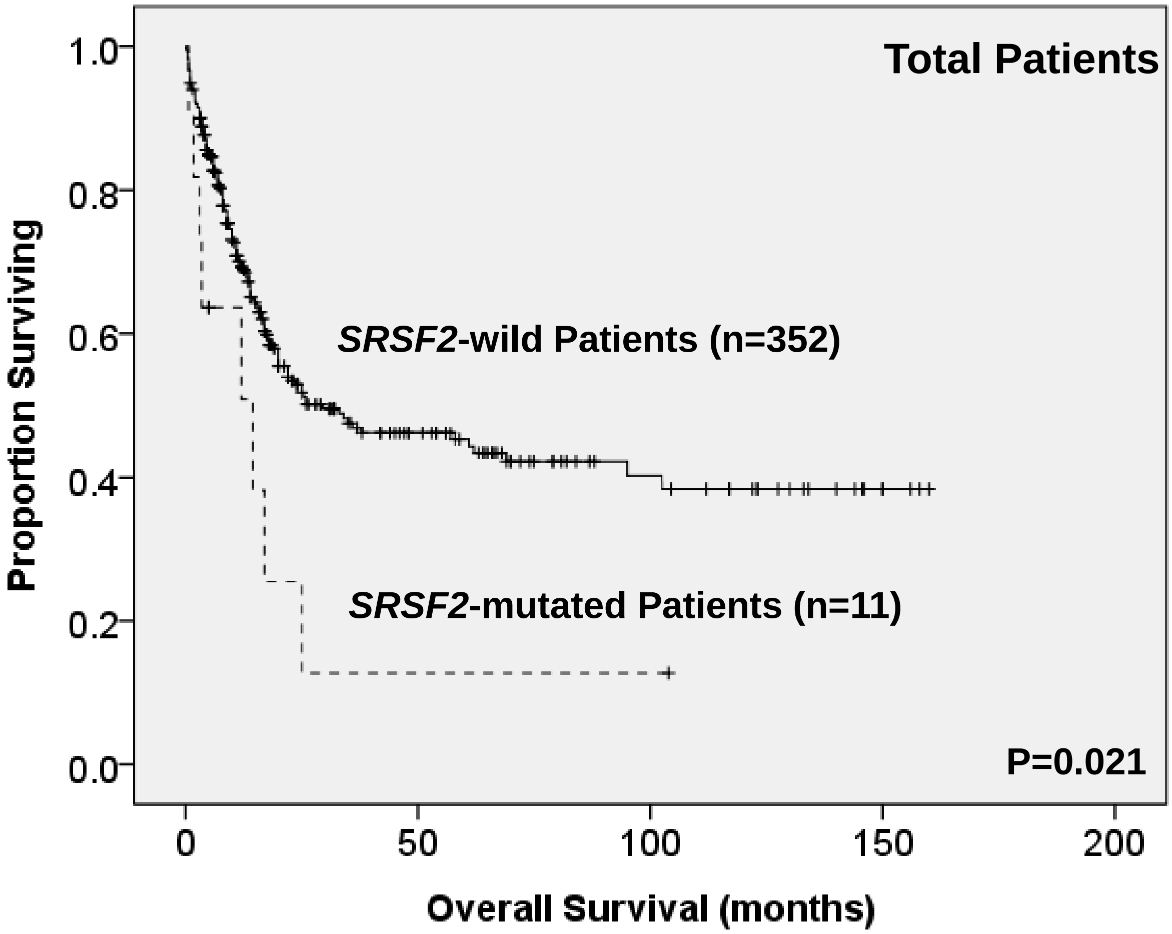

Supplementary Figure 1D

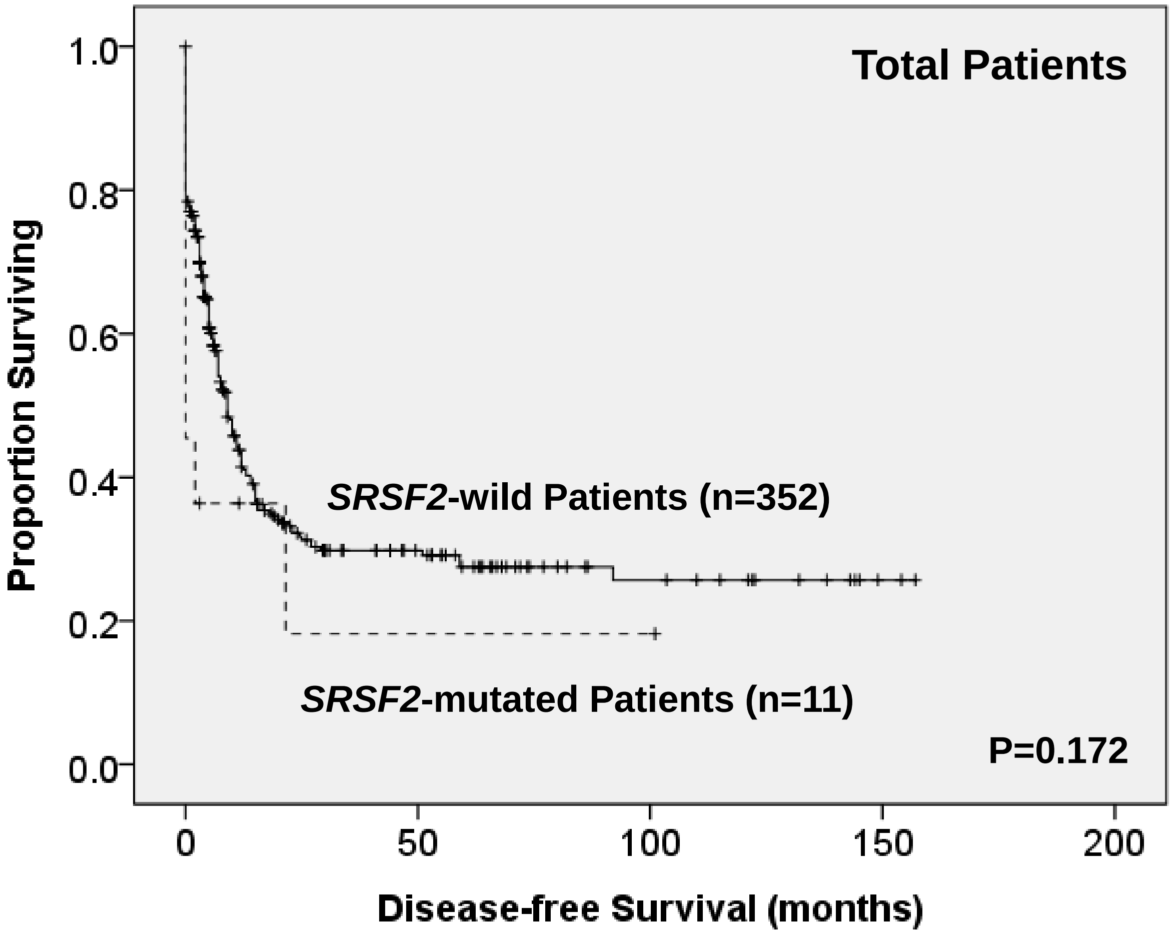

Supplementary Figure 1E

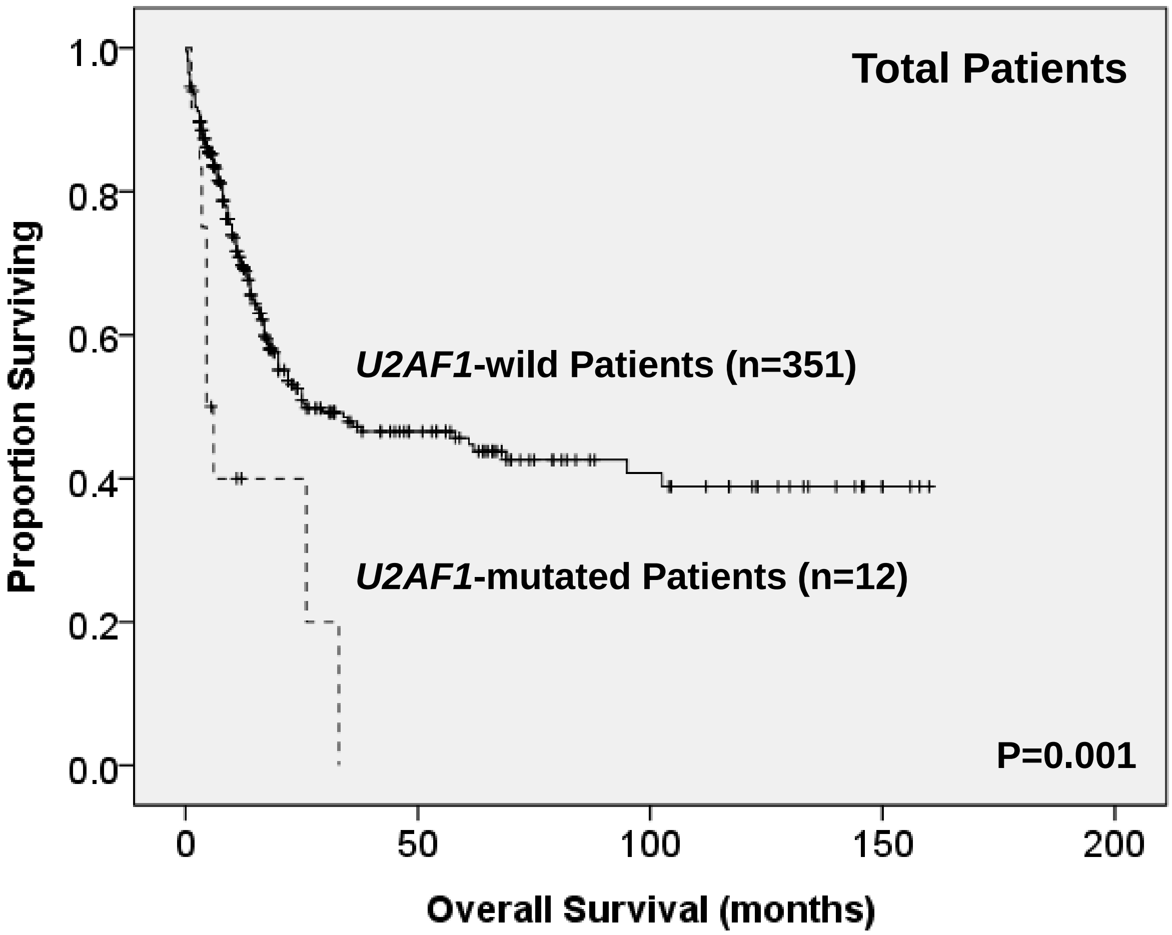

Supplementary Figure 1F

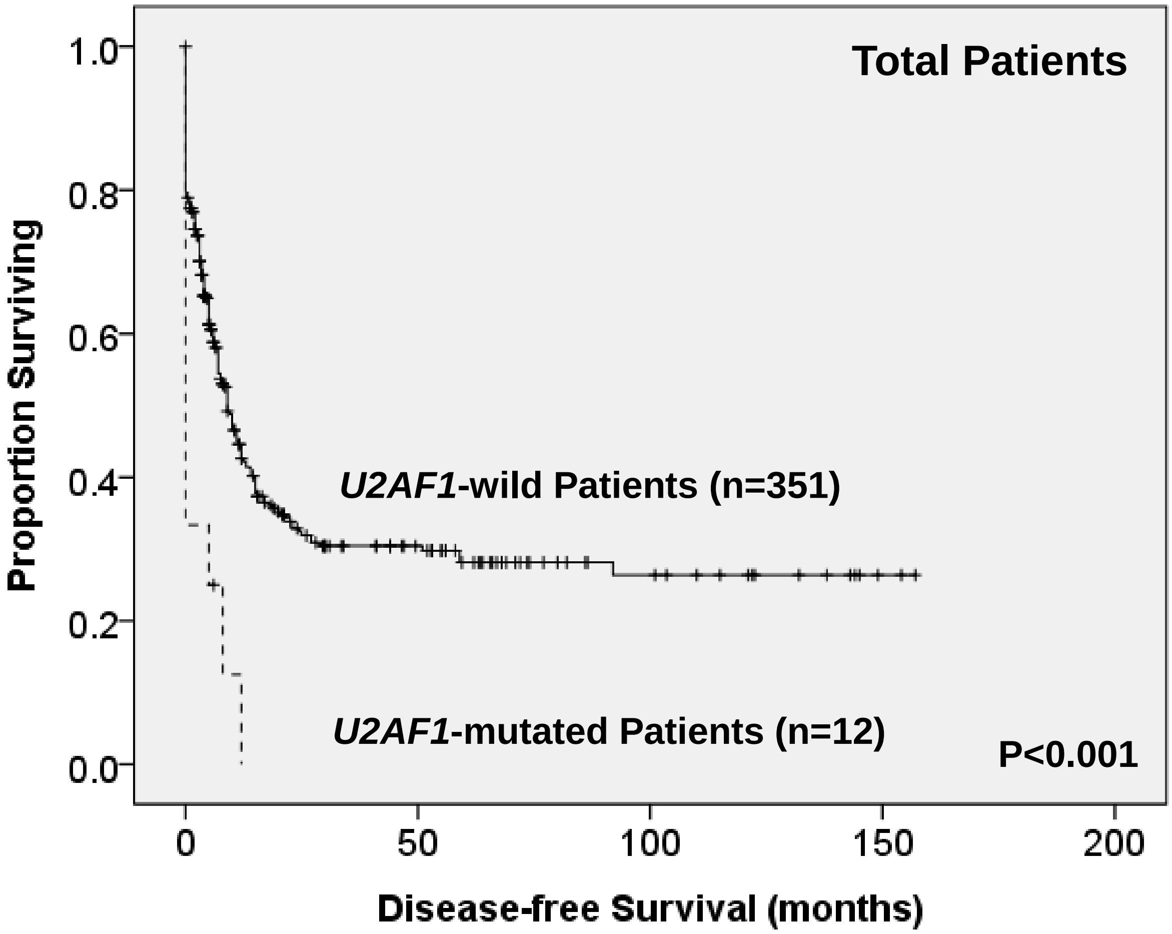

Supplementary Figure 2

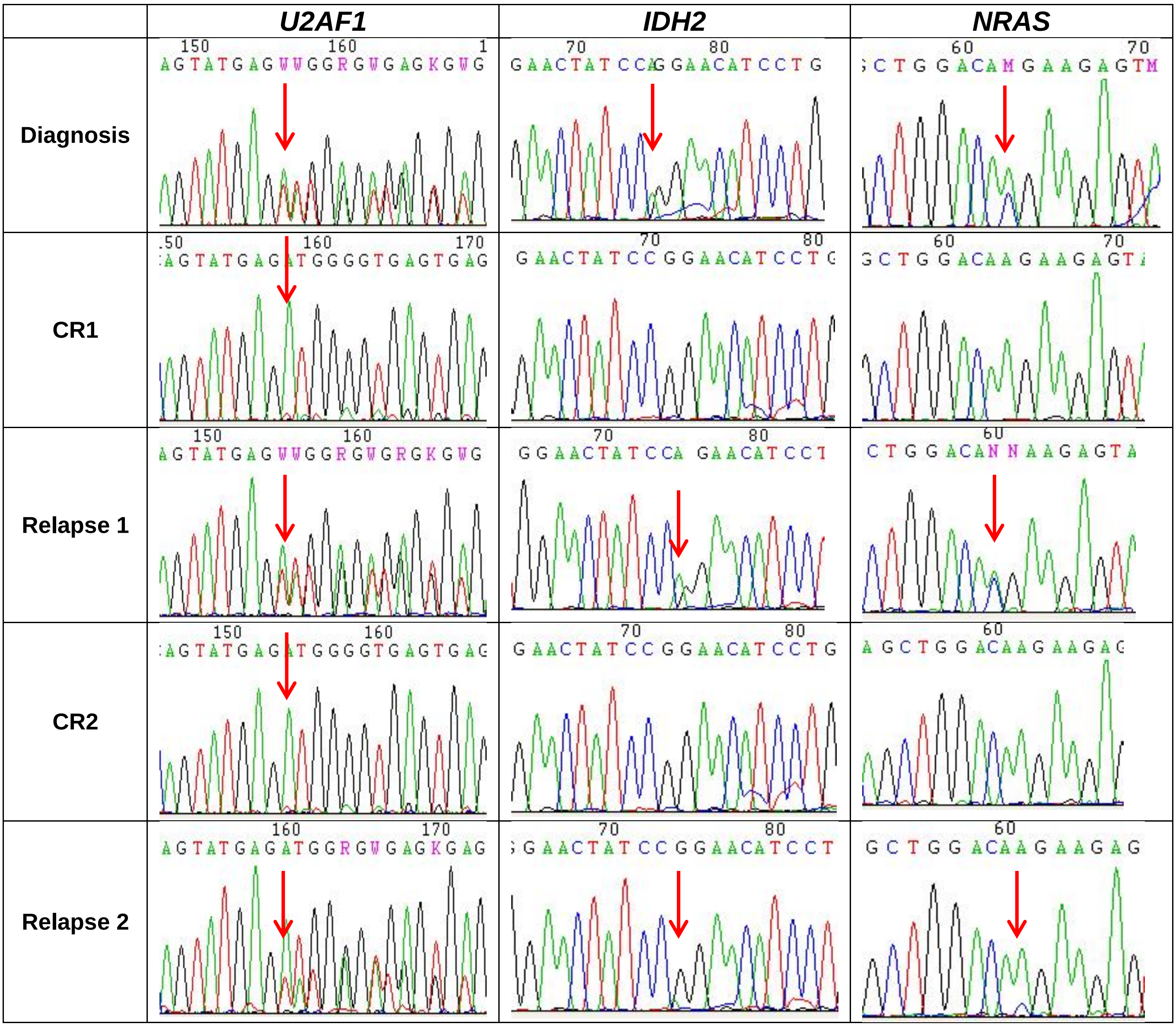

Supplement: Supplementary file 1 [file oncotarget-07-9084-s001.pdf]
